# New ICD-11 features for coding late sequelae and chronic post-procedural conditions

**DOI:** 10.1186/s12911-025-03121-5

**Published:** 2025-08-29

**Authors:** Danielle A. Southern, Bastien Boussat, Marie-Annick Le Pogam, William A. Ghali

**Affiliations:** 1 Centre for Health Informatics, Cumming School of Medicine, University of Calgary, Calgary, AB Canada; 2 Clinical Epidemiology Unit, Grenoble Alps University Hospital, Grenoble, France; 3 Department of Epidemiology and Health Systems, Center for Primary Care and Public Health (Unisanté), University of Lausanne, Lausanne, Switzerland; 4 Office of Vice President of Research & O’Brien Institute of Public Health, University of Calgary, Calgary, AB Canada

**Keywords:** International classification of diseases, Sequelae, Late effect, Post-procedural

## Abstract

There are many clinical circumstances in life where people live with chronic conditions (or states) that arose from either (1) a prior clinical diagnosis (e.g. a stroke) or (2) a prior healthcare-related event or medical procedure. Unfortunately, capturing such concepts is not straightforward in coded health data. This paper describes the coding rubric for sequelae (also often referred to as ‘late effects’) in the new ICD-11 coding system and some clinical coding examples. Earlier versions of ICD were constrained, in all but a few exceptions, by the need to combine all aspects of a clinical scenario into a single code. ICD-11 permits the clustering (postcoordination) of multiple codes to describe multifaceted clinical scenarios. This article features both precoordinated (single code) and postcoordinated (multi-code) descriptions of late effect situations where a prior health problem is the remote cause of current symptoms or conditions – i.e. sequelae. The late effect of a prior health problem rubric is yet another example of enhanced ICD-11 features that will improve future health information systems.

## Background

*A patient has joint contractures of several fingers from a previous burn injury.*

*A patient presents with a urethral stricture due to previous radiation for treatment of prostate cancer.*

There are many clinical circumstances where people live with chronic conditions (or states) that arose from a prior medical condition (e.g. a stroke) or a prior medical procedure. Sequelae can be aesthetic (e.g., scarring after a surgery), psychological (e.g., postpartum depression, PTSD), affect the functioning of organs or systems (e.g. liver fibrosis due to prolonged treatment with methotrexate), or be general (e.g., post-COVID-19 fatigue, central post-stroke pain). They can also be transient (e.g. a “whooping cough” lasting several weeks after remission of pertussis, a headache lasting more than a year after viral encephalitis, a transient pneumatocele after a bacterial lung infection, a transient skin depigmentation after an atopic dermatitis outbreak) or permanent (e.g. a lower limb amputation after severe trauma, post-tuberculosis bronchiectasis, post-stroke hemiplegia, a vegetative state after neurosurgery for treatment of a brain tumour) after the recovery from the original condition or procedure. Finally, sequelae can occur in the short term (e.g., < 1 months), medium-term (e.g., 1–5 months), or long term (e.g.,> 6 months) [1]. Despite such situations being commonplace, the capture and description of late sequelae and chronic post-procedural conditions are not straightforward in coded health data.

It is important to identify/capture sequelae in coded health data in order to describe the epidemiology of health consequences of diseases, injuries, poisoning, medical treatments, unhealthy behaviours and medical procedures and to study the causal relationship with the suspected prior medical/health condition or procedure. In addition, capturing sequelae can enable the adjustment of reimbursement systems for health care providers (as sequelae increase healthcare workload, costs, length of stay, tests and drug prescriptions, etc.) and patients (financial compensation systems for patients or 100% coverage by health insurance). Additional benefits of sequelae are enhancing diagnosis timing in coded data, improving casemix adjustment when comparing healthcare outcomes among providers or geographical areas, and assisting in planning health and social care infrastructure and resources.

This paper describes how to code for sequelae or late effects in the new International Classification of Diseases 11th version (ICD-11) coding system. We present clinical examples and use of online coding resources to demonstrate how these concepts would be coded in the new system.

### Coding of sequelae in ICD-10

In the International Classification of Diseases 10th version (ICD-10), sequelae concepts are sometimes captured in single codes where multiple clinical concepts are ‘precoordinated’ (bundled) into a single code (i.e., a lot of information wrapped into a single code). For example, I97.0 Postcardiotomy syndrome, K91.5 Postcholescystectomy syndrome, and N99.1 Postprocedural urethral stricture. These codes sit in body system chapters and are not comprehensive. This approach in a classification system is limited because there are many possibilities not captured in fully precoordinated concepts.

If there is not a precoordinated code, coders are instructed to first assign a code for the current condition under investigation. They are then instructed to select an additional code (placed in the immediately adjacent code field) from the T90-T98 category (Sequelae of injuries, of poisoning and of other consequences of external causes) as a diagnosis to identify the current problem as a sequela. Therefore, ICD-10 codes for each of the nature of the sequela and the underlying cause are coded side-by-side. This model has limitations. Those who generate the data need to know about this sequelae coding rule. Coders also need to know about the available code sets for sequelae. In addition, those who analyze the data need to know what different codes mean when sequelae are present, and they must be aware that in rare instances, adjacent codes must be interpreted as a single concept. In short, there is considerable potential for data errors, both in generation and analysis.

### ICD-11 coding of late sequalae

A newly applied feature of ICD-11 that supports combining (linking) two or more codes into a cluster is called postcoordination. Clustering and postcoordination, discussed in a previous article in this series [2], are the key to improving the richness of clinical information (in particular, the causal relationship and the chronology between the initial condition or procedure and the sequela). Postcoordination in ICD-11 allows for a clinical concept to be coded and reported to a greater level of specificity than was possible in ICD-10.

Despite this new feature, ICD-11 does have some precoordinated concepts that mirror those in ICD-10 (Table 1). For example, several sequelae of infectious diseases (e.g. 1G80 Sequelae of tuberculosis, 1G82 Sequelae of leprosy) and some chapter-specific sequelae (e.g. 8B25 Late effects of cerebrovascular disease and JB65 Sequelae of complication of pregnancy, childbirth, or the puerperium) have been brought forward from ICD-10. Given the multitude of conditions that can cause sequelae, completeness of precoordinated codes is not possible.

**Table 1:** Some examples of precoordinated concepts in ICD-10 and ICD-11

ICD-10	ICD-11
Sequelae of complication of pregnancy, childbirth, or the puerperium (O94)	Sequelae of complication of pregnancy, childbirth, or the puerperium (JB65)
Sequelae of tuberculosis (B90)	Sequelae of tuberculosis (1G80)
Sequelae of poliomyelitis (B91)	Sequelae of poliomyelitis (1G83)
Sequelae of leprosy (B92)	Sequelae of leprosy (1G82)
Sequelae of viral encephalitis (B94.1)	Sequelae of viral encephalitis (1G84)
Sequelae of trachoma (B94.0)	Sequelae of trachoma (1G81)
Sequelae of rickets (E64.3)	Sequelae of rickets (5B63)
Sequelae of cerebrovascular disease (I69)	Late effects of cerebrovascular disease (8B25)
Sequelae of protein-energy malnutrition (E64.0)	Sequelae of protein-energy malnutrition (5B60)
Sequelae of vitamin A deficiency (E64.1)	Sequelae of vitamin A deficiency (5B61)
Sequelae of vitamin C deficiency (E64.2)	Sequelae of vitamin C deficiency (5B62)
-	Sequelae of diphtheria (1G85)
-	Sequelae of other specified infectious diseases (1G8Y)
	Sequelae of unspecified infectious diseases (1G8Z)
-	Other specified sequelae of malnutrition or certain specified nutritional deficiencies (5B6Y)
-	Late effect of prior health problem, not elsewhere classified (QC50)

Fortunately, the generic code of QC50 Late effect of prior health problem, not elsewhere classified, unlocks the full potential of ICD-11 to fully capture late sequelae scenarios. The QC50 code can indicate that a prior health problem is now associated with a late effect causing current symptoms or conditions, allowing one to establish all possible combinations of codes and consider the diversity of the initial conditions. This concept excludes prior health problems *not* causing a current symptom or condition and *not* caused by the original injury. Formal coding rules have been developed for these circumstances [3]. Conditions documented as sequelae (or late effects) will typically be classified using postcoordination (with a few examples such as in Table 1). Further, the ICD-10 precoordinated categories for sequelae of external causes have been removed, and **sequelae will now be classified under the categories that best describe the original event**,** which will lead to an increase in the reported frequency of those specific categories.** Sequelae will now be indicated by a cluster identifying the sequelae condition, a code from Chap. 24 (QC50) and the original external cause code [3].

**Fig. 1:**
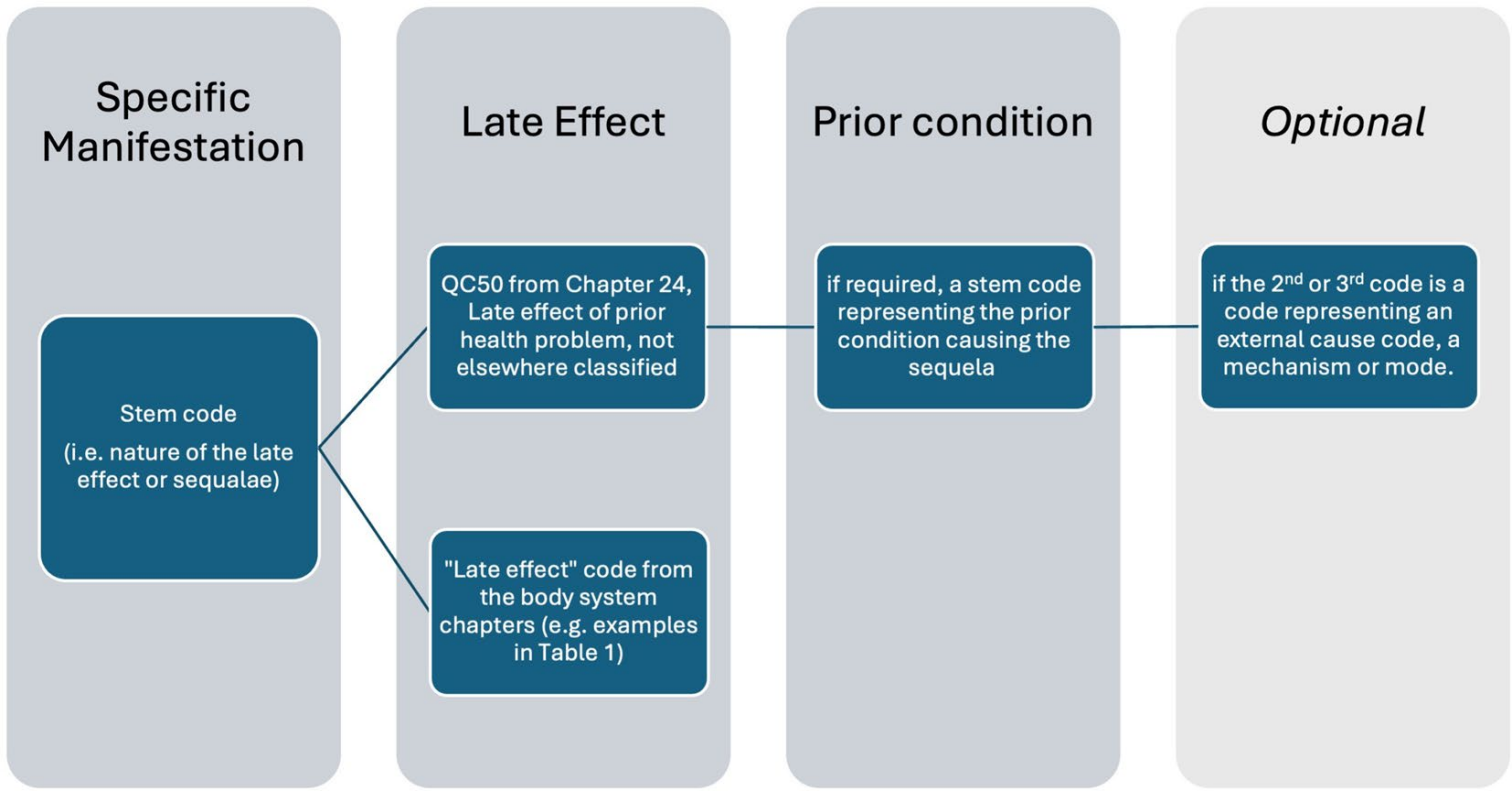
Cluster Elements

The cluster should contain (Fig. 1):

- First, a stem code identifying the specific manifestation (i.e. nature of the late effect or sequalae), and.
- Second, a stem code designating ‘late effect of’ (either a code from the body system chapters or the code QC50 from Chap. 24, Late effect of prior health problem, not elsewhere classified).
- Third, if using QC50 and if required, a stem code representing the prior condition causing the sequela.
- (optionally, and last, if the 2nd or 3rd code is a code representing an external cause code, a mechanism or mode.)

As noted above, ICD-11 provides specific categories when conditions are reported as sequelae, late effects, or other conditions (e.g. *1G80-1G8Y Sequelae of infectious diseases*). Many chronic clinical conditions occur either due to specific procedures and techniques or to removing an organ, e.g. postmastectomy lymphedema syndrome, post-irradiation hypothyroidism. In many instances, as in ICD-10, codes for such chronic postprocedural conditions are still available in ICD-11 within various body system chapters. For example, BE10 Postcardiotomy syndrome in Chap. 11 – Diseases of the circulatory system; 5D40.Z Postprocedural hypothyroidism, unspecified in Chap. 5 – Endocrine, nutritional or metabolic diseases; GC72 Postprocedural urethral stricture and GC70 Postoperative adhesions of the vagina in Chap. 16 – Diseases of the genitourinary system. By their very nature, these latter concepts are precoordinated codes that capture both the clinical condition and the notion of it being caused by a procedure. It is possible to use such codes alone without any clustering. However, coders can also use the 3-part model (as described in another paper in this series) with such codes to add specificity [6]. The model allows the addition of more specificity about the specific type of surgical procedure that caused the condition and the mode through which the procedure caused the condition.

### Coding examples

**Example 1:** Joint contracture present as a late effect of a prior burn.

- FA34.3 Contracture of joint.
- QC50 Late effect of prior health problem, not elsewhere classified.
- NE11 Burn of unspecified body region.
- Code: FA34.3/QC50/NE11.

**Example 2:** Hemiplegia present as a late effect of old cerebral ischemic stroke.

- MB53.Z Hemiplegia, unspecified.
- 8B25.0 Late effects of cerebral ischemic stroke.
- Code: MB53.Z/8B25.0.

#### Note

Some precoordinated concepts still exist for legacy reasons, as noted in Table 1. In this example, the concept of late effect and the underlying cause is already precoordinated in stem code 8B25.0.

**Example 3:** A Patient is admitted for rehabilitation training for paralysis of the left leg resulting from cerebral infarction three years ago.

- MB55.Z Monoplegia of lower extremity, unspecified (and an optional extension code to specify XK8G Left may be added).
- 8B25.0 Late effects of cerebral ischemic stroke.
- Code: MB55.Z&XK8G/8B25.0.

**Example 4:** Urethral stricture due to previous radiation for treatment of prostate cancer.

- GC72 Postprocedural urethral stricture.
- NF2Y Other specified injury, poisoning or certain other consequences of external causes (*index term: Sequelae of radiation*,* late effect of radiation*).
- PK81.C Radiation therapy associated with injury or harm in therapeutic use.
- PL11.Y Other specified mode of injury or harm associated with a surgical or other medical procedure.
- Code: GC72/NF2Y/PK81.C/PL11.Y.

In relation to the preceding examples, we reiterate that the overriding recommendation is that postcoordinated code clusters should be used whenever possible to code clinical detail as richly as possible. However, there will occasionally be instances where it is unnecessary to use postcoordinated code clusters because the code for the chronic postprocedural condition already contains full clinical detail.

In addition to chronic conditions that are sequelae of prior diseases, as in the examples above, post-procedural chronic conditions can also occur. **The following two examples highlight this type of situation involving sequelae**:

**Example 5:** Cataract lens fragments in eye following cataract surgery.

9D21 Cataract lens fragments in eye following cataract surgery (for this, it would be highly redundant to code “ophthalmic procedure” and a corresponding mode, given all the detail inherently embedded in this single code).

**Example 6:** Chronic radiodermatitis following radiotherapy.

EL61 Chronic radiodermatitis following radiotherapy (again, redundant to code “radiation therapy” as the procedure causing harm, and “mode unspecified” for this case).

**How things look in the ICD-11 coding tool and browser.**

**The ICD-11 Coding Tool** [4] **was developed as an open-access platform to support code searching and postcoordination. It can be used independently of vendor-specific software**,** and EHR vendors may choose to incorporate ICD-11 functionalities via the provided API. Importantly**,** the tool is designed to be widely accessible to all coders and health systems. WHO provides training resources** [5], **as adoption may require coder training**,** updates to local coding systems**,** and alignment with national health IT infrastructure.**

**Fig. 2:**
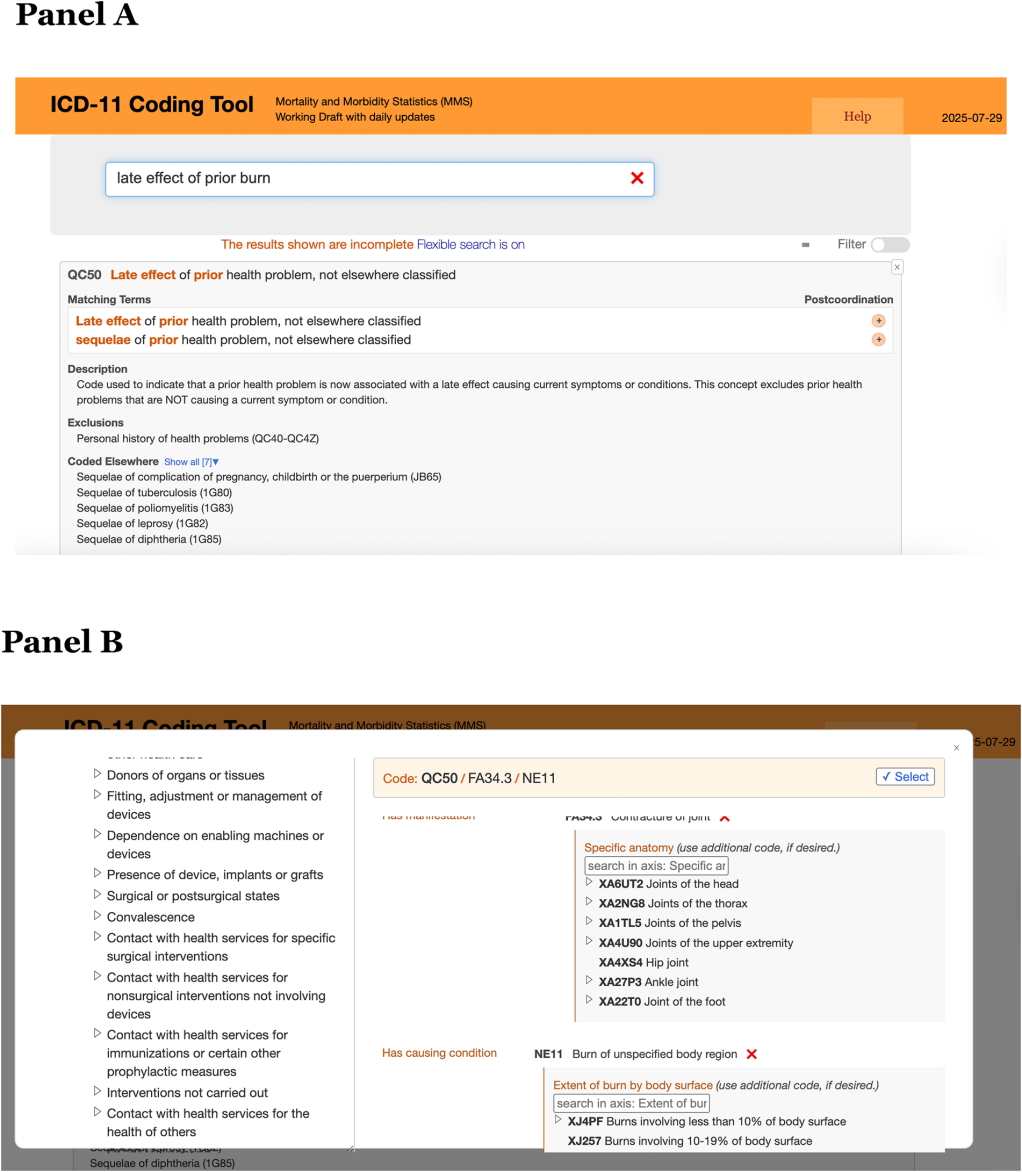
Example 1 coded using the Coding Tool (Panel **A**) and Browser (Panel **B**)

Fortunately, the ICD-11 online coding tools and browser help coders find the correct codes, and they prompt postcoordination when there are logical postcoordination options to consider. Figure 2 shows screenshots of the Coding Tool and Browser [6], demonstrating the use of options to create a postcoordinated code string. In addition, the figure illustrates the postcoordination features of the examples presented in Table 1 (Fig. 2: A screenshot of tooling relating to Example 2).

## Discussion

The coding of late effects of prior health conditions is yet another example of how and where ICD-11 is quite promising as a coding system. Postcoordination is the key that unlocks this useful coding mechanism. In the absence of precoordinated concepts, the QC50 code for late effects is essential for building the code cluster.

Just as with the three-part model for healthcare-related adverse events [7], there will be challenges in transitioning to a new coding system, and there may be a learning curve that coders and health systems will need to be overcome. In addition, training materials will be needed. Again, the WHO Reference Guide will help with this, as will the online Coding Tool features just mentioned.

It is, additionally, important for coders and analysts to understand the concept and challenges that follow. Data analysis algorithms need to be developed for these complex code strings; this will involve the development of analytic code that isolates portions of code strings to focus specifically on sequelae/late effect concepts. For example, if a patient has late effects (contracture) of a burn, the three-part model for late effects includes the prior (remote) diagnosis – in this example, a burn. If a researcher were performing an epidemiologic study on the proportion of burn injuries in a year, that report would be erroneous if exclusions of late sequelae diagnoses were not considered in the analytic code used to perform data analysis. This analytic caution applies to other ICD-11 concepts like ‘family history of’ (codes QC60 through QC6Z) and ‘Personal family history of’ (codes QC40 through QC4Z), and ‘ruled out’ diagnoses (code QA02). With these challenges in mind, the ability to capture late effects of a prior health problem lends to a far more promising information system than previous versions. It is important to remember that ICD-11 may have future use cases that extend beyond the traditional hospital discharge abstract. EMR systems might, for example, embed code clusters to describe clinical narratives in a codified manner.

In ICD-10, a sequela could be coded as a main condition with a different secondary code following it to note the sequela. In ICD-11, postcoordination allows for coding the sequela as the main condition with a single code cluster. Additionally, sequelae can be coded as secondary conditions for the stay.

Sequelae concepts can also be enriched further by complicated extension code descriptions of laterality and diagnosis timing [8]. In example 3, the paralysis of the left leg can be described with the extension code of XK8G. Additionally, because a sequela is an aftereffect of a disease, condition or injury once the initial cause is no longer present, the timing of sequelae can differ. Diagnosis timing extension codes can describe this timing (e.g. postoperative, developed after admission and present on admission). Lastly, as in example 4, the sequela concept can be further enriched by the additional postcoordination of the cause (radiation) and mode or mechanism (unknown in this case) of the sequela.

With the multitude of conditions, events and injuries that can cause sequelae, completeness of precoordinated codes is not feasible nor possible. With the new ICD-11 feature of postcoordination, rich descriptions of late effects of prior conditions, diseases or injuries are now able to be realized.

## Data Availability

Data sharing is not applicable to this article as no datasets were generated or analysed during the current study.

## Declarations

### About this supplement

This article has been published as part of BMC Medical Informatics and Decision Making Volume 21 Supplement 6 2021: Spotlight on ICD-11: New Features and New Opportunities. The full contents of the supplement are available at https://bmcmedinformdecismak.biomedcentral.com/articles/supplements/volume-21-supplement-6.

## Abbreviations

PTSD: Post traumatic Stress Disorder
COVID-19: Coronavirus Disease of 2019
ICD: International Classification of Diseases
ICD-10: International Classification of Diseases, 10th revision
ICD-11: International Classification of Diseases, 11th revision
